# The Water We Drink

**Published:** 1884-01

**Authors:** 


### THE WATER WE DRINK.

Some weeks ago I wrote a paper relating to the analysis of certain potable waters in and around this city.

In that article I stated that, so far as chemical analysis shows, there is no decided indication of pollution in our hydrant water, carefully filtered.

Since writing that article I have made a full and minute examination of the most important streams that supply the Water Works pond. The result of the investigation will, perhaps, be sufficiently interesting to the people to warrant its publication.

I first explored the head streams of Todd's branch, rising in Poplar Spring, and a swamp flat near the defunct ice factory, both springs being situated just south of the Central Railroad. Besides these permanent sources of water, this branch gets a large accession of washings in wet weather from the adjacent corner of West End and from the east side of the old United States Barracks, including between them a large and thickly settled area on both sides of Peters street. One sluice of this drainage passes under the Central Railroad just a little south of the ice Factory, and another quite as large under the same road and Whitehall street in the bottom between McMasters' and Whitehall street bridge over the E. T & Va. R. R. It is this sluice which drains the backyards, stables, kitchens and privies of East Barracks.

Now, from the top of an embankment near the bridge on Whitehall street, just mentioned, let us take a view of the hills and valley washed by Todd's branch.

This picturesque region constitutes now one of the busiest and most densely populated outer areas of Atlanta. It embraces all the land, with its innumerable dwellsngs, kitchens, cow-houses, stables and privies, which extends from the sluice designated, near the factory, to the corner of McDaniel street and the Central Railroad on one side and on another from this corner, across Whitehall street, by Wells' corner to a point scarcely beyond the Wadley Row on McDaniel street.

Here the water-shed between the branch and the streams that run southeast of the city deflectst to the right or southeast, and runs directly to the E. T., Va. & Ga. machine shops. On the hill slopes and valleys in the centre and along Todd's branch on the margin of this area, stands Mechanicsville, the Timbuctoo of Atlanta. The huts here are huddled close together, affording a large accumulation of filth between them, which is never removed, except when washed by the rains into the branch, while the privies stand very near to or immediately on the stream. When these become clogged with matter, it is quickly dumped into the passing water as it goes on its mission of supplying, from the Water Works pond, a wholesome beverage for the people of the city.

It is true that in many, perhaps most of the houses, it passes through a good filter before being used, and it then gives a colorless transparency in the glass, and is quite palatable; but it is well known that while a filter may remove all suspended impurities, it does not take out dissolved pollution, nor a single germ of any zymotic disease, such as typhoid fever and diphtheria. This is sufficiently seen in the fact that diseases of the most serious nature are capable of being transmitted even by milk of cows having access to sewer water filled with the germs of such diseases—notably of diphtheria, typhoid fever and scarlet fever.

The filter will not only decolorize the water, making it clear and limpid, but will deoderize it as well, yielding a sample of water as sweet and palatable as that from any mountain stream, and yet the living organisms of deadly disease are still reveling there, defying the most delicate chemical analysis, and often the highest powers of the microscope.

In this view of the situation, what will Atlanta do about it?—rather, what will the city fathers do about it? It will not pass muster with the people, as heretofore, to pooh, pooh this matter as a false alarm, gotten up for the sake of a little newspaper notoriety, if not from some worse motive. It is hardly possible for a writer who is competent to make such an investigation, to trifle in regard to facts of so serious a nature. If there are any doubting Thomases in the Council or on the Board of Health, let them join the editor of the ATLANTA MEDICAL REGISTER, some pleasant afternoon, in a stroll down Todd's branch, from its source, near the Central Railroad, to its debouch into the Water Works pond. It will be better and more satisfactory for the investigation, to defer the stroll till some hot day next summer, when heat will be present in sufficient force to prepare things for the regalement of the olfactories as well as for the eye. The party can then take in the situation by more than a single sense. It may require all this, and more, to convince some of the existence of the facts.

Is there any remedy for the difficulty with the present system of water supply? None in the power of man, unless, by a decree of the Council, a large and important part of the city be permanently laid waste, or Todd's branch turned forever, by a deep tunnel under its water-shed, from the Water Works pond by another channel to the waters of the Ocmulgee.

The question of wholesome water supply for the the city is made still more serious and urgent by the fact that her potable wells are either fast drying up or becoming so polluted as to be nauseous and dangerous in the last degree. As the close pavement of the streets extends, and a larger area of the city is covered by houses, the less becomes the supply of water draining into the wells, and the more polluted the water which does reach them.

One thing is certain, that in a very short time the men who may have in charge the sanitation and well-being of the city of Atlanta will be called upon to solve a practical question, which, in comparison with the late cow law or the retailing of liquor, will be as the tunneling of the Alps to the sweeping of the gutters.

